# The Effects of Interactive eBooks on Dyspnea Assessment and Management among Emergency Medical Technicians: A Repeated-Measures Analysis

**DOI:** 10.3390/healthcare10101932

**Published:** 2022-10-01

**Authors:** Chiu-Kuei Sung, Chien-Lin Kuo, Jen-Tse Kuan

**Affiliations:** 1 Department of Nursing, West Garden Hospital, West Garden Health Care Group, Taipei City 108, Taiwan; 2 Department of Allied Health Education & Digital Learning, National Taipei University of Nursing and Health Sciences, Taipei City 112, Taiwan; 3 Department of Emergency Medicine, Chang Gung Memorial Hospital (Linkou Branch), Taoyuan City 333, Taiwan

**Keywords:** emergency medical technician, interactive eBook, dyspnea, healthcare education

## Abstract

Dyspnea is a common emergency requiring urgent care, and a variety of factors may mislead emergency medical technicians (EMTs). Typically, EMT education uses traditional lectures with paper books. The effect of interactive eBooks on EMT learning has not been explored. This study aims to develop an interactive eBook in dyspnea assessment and management and to evaluate its learning effect. A quasi-experimental design with three repeated measures was used. A total of 117 EMTs were recruited and assigned to the experimental group (eBook, n = 56) and the comparison group (paper book, n = 61). Questionnaires were administered to both groups at three time points. The results show that both materials can improve cognition and that the interactive eBook has better effects than the paper book. The interactive eBook motivated EMT to learn more than the paper book, but motivation did not last for five weeks. The long-term effect of the interactive eBook on confidence compared to the paper book is significant. The eBook can include real cases, concept maps, videos, quizzes, and instant feedback to meet learner needs. Health educators could integrate technology and cognitive strategies into EMTs’ training curricula to improve their ability to provide better emergency medical services.

## 1. Introduction

The number of ambulances dispatched in Taiwan has increased year by year, highlighting the strong demand for emergency medical services (EMS) [1]. The lives and safety of patients depend on the quality of EMS they receive before arriving at the hospital. The assessment and management abilities of emergency medical technicians (EMTs) affect EMS quality. Taiwanese EMS rely on training organizations to issue licenses to practice. The EMT Administration Regulation sets the number of training hours and curricula topics for the three levels of EMT. There is no national EMT education standard for EMT instructors as a guide for training and curriculum design [2]. Typically, the EMT-1 curriculum is 40 h long and focuses on first aid techniques such as assessing vital signs, monitoring blood oxygen concentrations, removing foreign bodies from the airway, providing oral-nasal ventilation, and maintaining an open airway and oxygen supply. The EMT-2 curriculum is 280 h long, and in addition to the contents of EMT-1, it also includes the use of advanced airway and inhaled bronchodilators [3]. In addition, traditional paper books combined with lectures are common teaching materials.

Due to their non-medical background and limited training hours, most EMTs have insufficient medical science knowledge to handle various emergency situations and implement appropriate measures. Moreover, EMTs have an ability gap in dyspnea assessment and decision-making regarding respiratory treatment [4]. EMTs’ self-confidence in their own ability is also affected by noise from people on site or concerns about medical disputes, prompting EMTs to provide emergency services. Therefore, it is imperative to redesign the teaching of this topic.

There are no studies that have reviewed or discussed teaching methods in Taiwanese EMT training. eBooks have begun to be utilized in schools in the last two decades. eBooks can embed interactive elements such as pictures, videos, sound, and interactivity to attract learners’ interest [5]. Compared with traditional paper reading, the use of an interactive eBook is perceived as helpful for students to learn in a more convenient, efficient, and extensive way [6]. A systematic review study compared reading performance between e-texts with interactive features and paper/static e-texts. Based on the meta-analyses of 26 studies, it found that interactive e-texts benefited reading performance compared to paper texts (*p* < 0.001) [7]. Since eBooks are easy to operate by course planners and learners [8], they have been applied in health professional education in recent years and have achieved positive learning outcomes [9,10,11]. However, the effect on EMT has not been studied.

Concept maps (CMs) are graphical tools that represent the meaning of a set of concepts organized into propositions. Concept mapping is an effective strategy for knowledge retention and transfer. Learners connect learned knowledge with pre-acquired knowledge to form and organize new knowledge, and connections are labeled and presented as hierarchies in the network [12]. The application of CMs helps learners to classify and combine structures and form visual memory, thereby avoiding visual confusion, reducing cognitive load and enhancing critical thinking ability [13]. To improve the ability of EMT in dyspnea assessment and management, this study aimed to develop an interactive eBook combining CM strategies and to evaluate its effects on motivation to learn, cognition, and confidence.

## 2. Materials and Methods

### 2.1. Study Design

This study adopted a two-group quasi-experimental design which involved repeated measurement at three time points, a pretest (T1, before course), posttest (T2, one week later), and follow-up test (T3, five weeks later).

The number of samples was determined using G*Power 3.1.9.7 (effect size = 0.25, α = 0.05, power = 0.9), which resulted in a sample size of 116. The inclusion criteria were (1) EMT-1 credential holders and (2) the completion of a 4 h course of dyspnea assessment and management. EMTs from the 2019 fire prevention program were invited to participate in this study. They were assigned according to training class number, with odd numbers assigned to the experimental group (EG) and even numbers assigned to the comparison group (CG). The EG used the eBook, while the CG read the paper book for self-learning.

### 2.2. Design of the Interactive eBook

**Figure 1:**
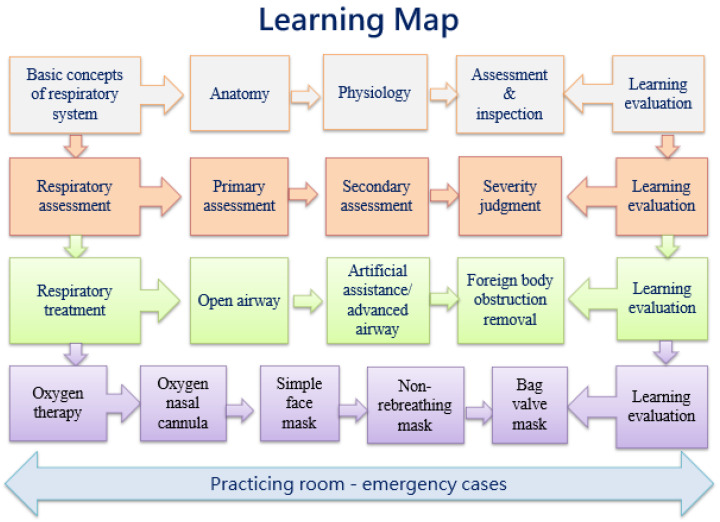
Learning map interface of the interactive eBook.

**Figure 2:**
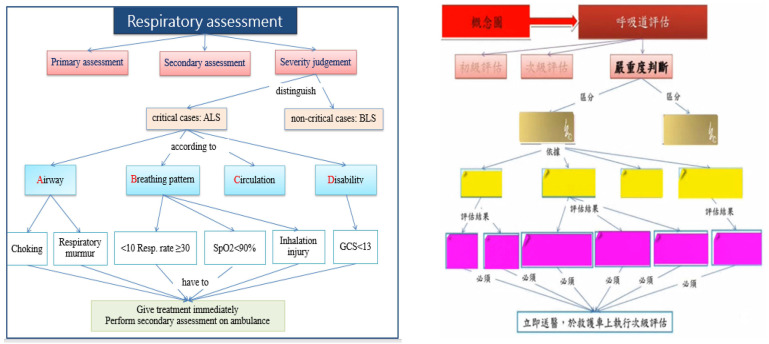
Concept map in the interactive eBook, right: before scratch-offs, left: after scratch-offs.

The interactive eBook was designed based on information processing theory [14], combining case-based learning (CBL) and CM strategies. The researchers used the software of SimMAGIC (Hamastar, Kaohsiung, Taiwan) to edit the eBook, which included some interactive features, such as highlights, cover-ups, scratch-offs, bookmarks, and prompt feedback for preset questions. The learners can take the quizzes and gain instant feedback after each unit of study. The learning map consists of five units: basic concepts of the respiratory system (anatomy and physiology), airway assessment, airway treatment, oxygen therapy and emergency cases of dyspnea (e.g., asthma, hyperventilation, suppressive lung disease, carbon poisoning, cardiorespiratory failure) (Figure 1). Selected video clips are embedded to help learning, such as the movement of the diaphragm as it rises and falls during breathing. Additionally, color-coded CMs are provided in the eBook to break down complex information into multiple CMs (Figure 2).

### 2.3. Instruments

The research tools used in this study included four parts: a demographic data sheet, motivation for learning, cognition test, and confidence scale. Five experts in emergency medicine, emergency dispatch, respiratory therapy, and nursing education were invited to build the expert validity of the teaching materials and questionnaires. The revised version was then pre-tested by 30 EMTs. The time required to complete the questionnaires is approximately 30 min.

#### 2.3.1. Demographic Data Sheet

The demographic data included gender, age, education, experience of emergency treatment, and experience of using the eBook. These data were collected at T1 only.

#### 2.3.2. Motivation for Learning

The scale derived from Wang and Chen [15] contained five items and was scored on a five-point Likert scale (1 = strongly disagree, 5 = strongly agree). Higher scores indicated stronger motivation to learn. The content validity index (CVI) was 1.0, and the Cronbach’s α was 0.85.

#### 2.3.3. Cognition Test (Version A, and B)

The self-developed scale comprised fifteen multiple-choice questions on the dyspnea assessment and management. The total score ranged from 0 to 15, 1 point for correct answers, no points for incorrect or incomplete answers. The higher the score, the higher the cognition level. To avoid the effect of repeated measures, two versions of the scale were developed. The CVI of the scale was 1.0, and the Cronbach’s α for version A and B was 0.81 and 0.83, respectively. Version A was used at T1 and T2, while Version B was used at T3.

#### 2.3.4. Confidence in Dyspnea Assessment and Management

The scale originating from Cherng [16] consisted of ten items and was scored on a five-point Likert scale (1 = never, 5 = always). A higher score indicated a higher level of self-confidence. The CVI of the scale was 1.0, and the Cronbach’s α was 0.87.

### 2.4. Procedures

The study was approved by the institutional review board (IRB) of Taipei City Hospital (#TCHIRPB-10802006-E). After obtaining IRB approval, the researchers contacted the administrator of the fire department and explained the study purpose and procedures to the EMTs. Before starting the course, a pre-test questionnaire was distributed to EMTs who agreed to participate in the study. Both groups were taught by the same teacher and the teaching methods include lectures, drills, and hands-on activities. After the 4 h classroom instruction, supplementary materials were provided to participants for self-learning. The EG downloaded the interactive eBook onto their smartphone or tablet, while the CG received the paper book. One week later, a post-test (T1) questionnaire was given, and then a follow-up test (T2) was conducted 5 weeks later. Finally, the eBook was shared with the CG after the follow-up test.

### 2.5. Data Analysis

The authors employed SPSS for Windows for data analysis. Participants’ characteristics were analyzed using means and standard deviations for continuous variables, and frequencies and percentages for categorical variables. The Mann–Whitney U test and Kruskal–Wallis test were used to examine the demographics between groups. ANCOVA was performed to compare means between groups with pretest as the covariate. A generalized estimating equation (GEE) model was used for the analysis of repeated measurements and the group, time, and interaction effects. All results with *p* < 0.05 were considered statistically significant.

## 3. Results

### 3.1. Participant Characteristics

A total of 117 EMTs completed three measurements with a loss rate of 1.7%. The majority of participants were male (91.5%), with an average age of 26.27 ± 2.94 years. More than half of them had a bachelor’s degree or higher. The majority had a non-medical educational background (95.7%) and had no experience in emergency care (81.2%). Most of them had never heard of interactive eBooks (67.5%) and only 9.4% had experience of using eBooks. The two groups are homogenous in demographic characteristics (Table 1).

### 3.2. Effects on Motivation, Cognition, and Confidence

Table 2 shows the posttest scores of motivation, cognition, and confidence for both groups. Levene’s test (*p* = 0.139, 0.197, 0.500) for equality of variances indicated that the assumption of the homogeneity of variances across groups was met. ANCOVA was then performed, and after excluding the effect of the pre-test (T0), it was found that there were significant differences between the two groups in motivation (F = 3.95, *p* < 0.05) and cognition (F = 29.62, *p* < 0.01) at T1. The results reveal that the interactive eBooks outperformed the paper book in improving motivation and cognition.

The retention effect (T2) of the intervention is shown in Table 3. There was homogeneity of variances, as assessed by the Levene’s test for equality of variances on follow-up test (*p* = 0.072, 0.803, 0.078). ANCOVA was then conducted, and after excluding the effects of the pre-test (T0), the EG outperformed CG in cognition (F = 5.96, *p* < 0.05) and confidence (F = 7.94, *p* < 0.01). The results show that the long-term effects (T2) of the interactive eBooks were better than the paper books in terms of cognition and confidence

**Figure 3:**
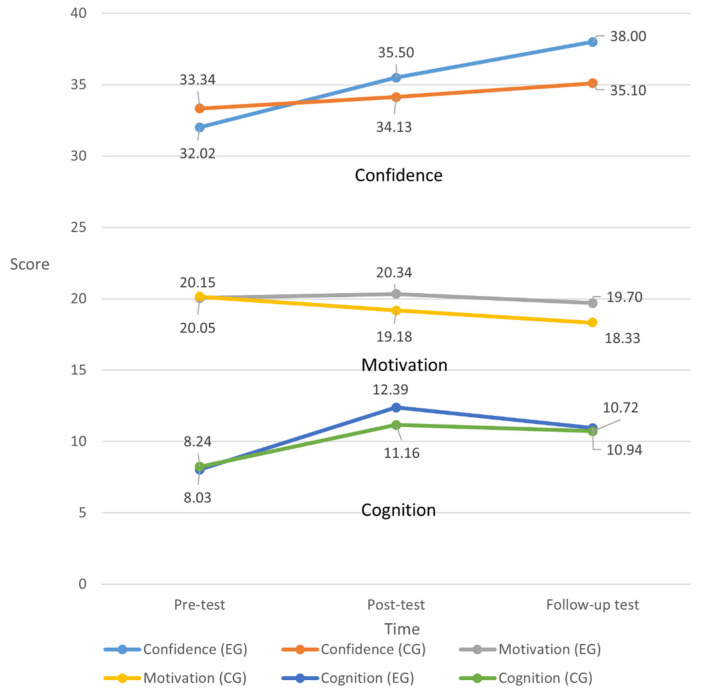
Differences in scores of confidence, motivation, and cognition between the two groups on the three tests (EG = experimental group; CG = comparison group).

A GEE model was used to compare differences in improvement. The learning effect of the intervention is shown in Table 4 with the pre-test (T0) as the benchmark. The motivation scores declined at T2 (β = −1.82, *p* < 0.01). The cognition scores increased at T1 and T2 (β = 2.92, 2.48, *p* < 0.001). The change in cognitive scores from T1 to T0 in EG was 1.44 points greater than in CG (β =1.44, *p* < 0.001). The change in confidence scores from T2 to T0 in EG was 4.23 points greater than in CG (β = 4.23, *p* < 0.01). Figure 3 shows the change in scores at the three measuring time points. These results reveal that the interactive eBook was superior to the paper books in enhancing cognition and confidence. However, the learning motivation in both groups could not be maintained at T2.

## 4. Discussion

This is the first study to evaluate the effects of the interactive eBook as a supplementary material on EMT to determine whether eBooks can improve their motivation, cognition, and confidence regarding dyspnea assessment and treatment. The interactive features of the eBook make learning more dynamic, overcoming the one-way and tedious nature of learning, and thus enhances the motivation to learn. Most of the participants are first-time users of interactive eBooks. The novelty of multimedia technology and interaction greatly enhances their motivation. Over time, however, the novelty wore off and participants’ motivation declined. The result of this study is consistent with a previous study [9]. Ko [17] suggested that interactive eBooks were more motivating than paper materials, and thus foster self-learning abilities. However, Chuang [18] applied multimedia materials and an immediate response system to teach physiology. He examined 120 nursing students and found that their learning motivation continued to grow on follow-up tests.

The supplementary materials used in this study all increase the knowledge of EMT, and the interactive eBooks were superior to the paper books. However, the memory of the course content faded after five weeks, resulting in a slight drop in cognition scores. The results of this study partially support previous studies [6,8,19]. Liu [6] argued that for content retention, paper- and ebook-based study demonstrate no significant differences. Interactive eBooks effectively deliver information, enhance reading comprehension and self-efficacy, and reduce learning frustration. eBooks can embed common dyspnea cases that allow learners to grasp the emergency situation, reduce the gap between theory and practice, and improve their ability to apply what they have learned [20,21]. Case-based learning is useful for presenting complex information or solving problems [22]. CBL can offer EMTs opportunities to practice and apply their knowledge to real cases before they start work in the field [2]. Concept mapping was another effective comprehension strategy. Repeated reading in five weeks helps learners to understand and remember complex and abstract concepts. Learners can also solve problems through critical thinking exercises [23,24]. The use of CBL and CM strategies facilitates the organization and integration of knowledge while promoting active inquiry-based learning for students beginning clinical practice [25]. Combining multimedia with CMs can reduce learners’ cognitive load and improve learning performance [26]. On the other hand, Rockinson-Szapkiw et al. [27] and Stirling and Birt [5] argued that interactive eBooks can engage learners’ interest and improve their skill performance rather than improving their comprehension and even increasing cognitive load. Lim et al. [28] compared the effects of interactive eBook and paper-based reading on high school students’ reading comprehension. They claimed that interactive features not designed to aid understanding can distract students from a reading task, which may hinder their comprehension.

Based on the study findings, the interactive eBook improved learners’ confidence more than the paper books. Additionally, confidence continued to increase after five weeks. These results are consistent with previous studies [6,9,29]. Chuang et al. [9] stated that skills demonstration videos via smartphones, interactive eBooks or DVDs can increase students’ confidence in practice skills. Learners can repeatedly practice unfamiliar assessment and management procedures, gain experience, and apply what they have learned into practice, developing professional judgment and confidence [11,21]. The dyspnea cases presented in the interactive eBook were drawn from authentic emergency situations. Real scenarios enable EMTs to learn in a simulated environment and take advantage of multimedia benefits such as control, practice, instant feedback and interactivity.

### Research Limitation

The subjects were purposively recruited from an EMT training program in Taoyuan city, which may limit the generalizability of the findings. During the study period, a typhoon struck, and the follow-up test was delayed by a week. This also coincides with the final exam of the EMT-2 training course. As a result, subjects had to take both the exam and the study measurements, creating a burden of filling out questionnaires that could affect the study results. Moreover, even though the researchers reminded the participants weekly to read supplementary materials, it is hard to know how well they were using eBooks outside of the classroom. Future studies may examine which interactive features are most helpful for learning through eBooks.

## 5. Conclusions

This study shows that both teaching materials can improve cognition, and that the interactive eBook has better effects than the paper book. The interactive eBook increases motivation more than the paper book, but motivation did not last for five weeks. The long-term effect of the interactive eBook on confidence compared to the paper book is significant. Additionally, EMTs favored the eBook and were satisfied with using it for self-learning.

### Practical Implication

The present learning environment is a blend of the physical classroom and online learning media. Compared to paper books, interactive eBooks are easier to update and allow for the tailoring of content to the specific needs of the course, students or instructors. Health educators could refine the EMT training curricula by integrating technology with cognitive strategies to improve EMTs’ abilities to provide better prehospital care services. The design of interactive eBook can include real case scenarios, concept maps, videos, prompts, questions, and instant feedback to improve EMTs’ learning outcomes.

## Figures and Tables

**Table 1:** Demographic characteristics (N = 117).

Variable	CG (*n* = 61) *N* (%)	EG (*n* = 56) *N* (%)	Z/*χ*^2^	*p*
Gender ^a^			0.518	0.604
female	6 (9.8)	4 (7.1)		
male	55 (90.2)	52 (92.9)		
Age ^b^			0.156	0.692
20~24	19 (31.1)	18 (32.1)		
25~30	34 (55.7)	33 (58.9)		
>30	8 (13.1)	5 (8.9)		
Education ^b^			1.022	0.312
High school	9 (14.8)	5 (8.9)		
Associate degree	13 (21.3)	10 (17.9)		
Bachelor	33 (54.1)	35 (62.5)		
Master	6 (9.8)	6 (10.7)		
Medical educational background ^a^			−1.564	0.118
No	57 (93.4)	55 (98.2)		
Yes	4 (6.6)	1 (1.8)		
Experience of emergency care ^a^			−0.222	0.825
No	50 (82)	45 (80.4)		
Yes	11 (18)	11 (19.6)		
Heard of eBook ^a^			−0.320	0.749
No	42 (68.9)	37 (66.1)		
Yes	19(31.1)	19(33.9)		
Experience of using eBook ^a^			−0.167	0.867
No	55 (90.2)	51 (91.1)		
Yes	6 (9.8)	5 (8.9)		

Notes: EG = experimental group; CG = comparison group; ^a^ Mann–Whitney U test; ^b^ Kruskal–Wallis test.

**Table 2:** Summary of ANCOVA on the post-test of motivation, cognition, and confidence between two groups.

Group	n	Mean	S.D.	Adjusted Mean	Std. Error	F Value	*p*
Motivation						3.95	0.049 *
EG	56	20.34	2.67	20.34	0.40		
CG	61	19.18	3.49	19.20	0.42		
Cognition						29.62	0.000 **
EG	56	12.39	1.33	12.44	0.17		
CG	61	11.16	1.56	11.12	0.17		
Confidence						1.50	0.224
EG	56	35.50	5.97	35.54	0.85		
CG	61	34.13	6.58	34.10	0.81		

Notes. EG = experimental group; CG = comparison group; * *p* < 0.05, ** *p* < 0.01.

**Table 3:** Summary of ANCOVA on the follow-up test of motivation, cognition, and confidence between two groups.

Group	n	Mean	S.D.	Adjusted Mean	Std. Error	F Value	*p*
Motivation						5.96	0.016 *
EG	56	19.7	2.52	19.69	0.402		
CG	61	18.33	3.4	18.33	0.386		
Cognition						1.71	0.194
EG	56	10.95	1.79	11.01	0.193		
CG	61	10.72	1.71	10.66	0.185		
Confidence						7.94	0.006 **
EG	56	38	5.31	38.07	0.778		
CG	61	35.1	6.27	35.03	0.745		

Notes. EG = experimental group; CG = comparison group; * *p* < 0.05, ** *p* < 0.01.

**Table 4:** GEE analysis of intervention effect on motivation, cognition, and confidence.

Parameter	Motivation		Cognition		Confidence	
	β	*p*	β	*p*	β	*p*
Intercept	20.15	<0.001 ***	8.25	<0.001 ***	33.34	<0.001 ***
Treatment Group (EG vs. CG)	−0.09	0.881	−0.21	0.492	−1.33	0.316
Time (T2 vs. T0)	−1.82	0.006 **	2.48	<0.001 ***	1.75	0.084
Time (T1 vs. T0)	−0.97	0.141	2.92	<0.001 ***	0.79	0.518
Interaction EG × (T2 vs. T0)	1.46	0.092	0.44	0.129	4.23	0.008 **
Interaction EG × (T1 vs. T0)	1.25	0.158	1.44	<0.001 ***	2.70	0.111

Notes. GEE = generalized estimating equation; EG = experimental group; CG = comparison group; T0 = Pretest; T1 = Posttest; T2 = Follow-up test; ** *p* < 0.01, *** *p* < 0.001.

## Data Availability

Not applicable.
